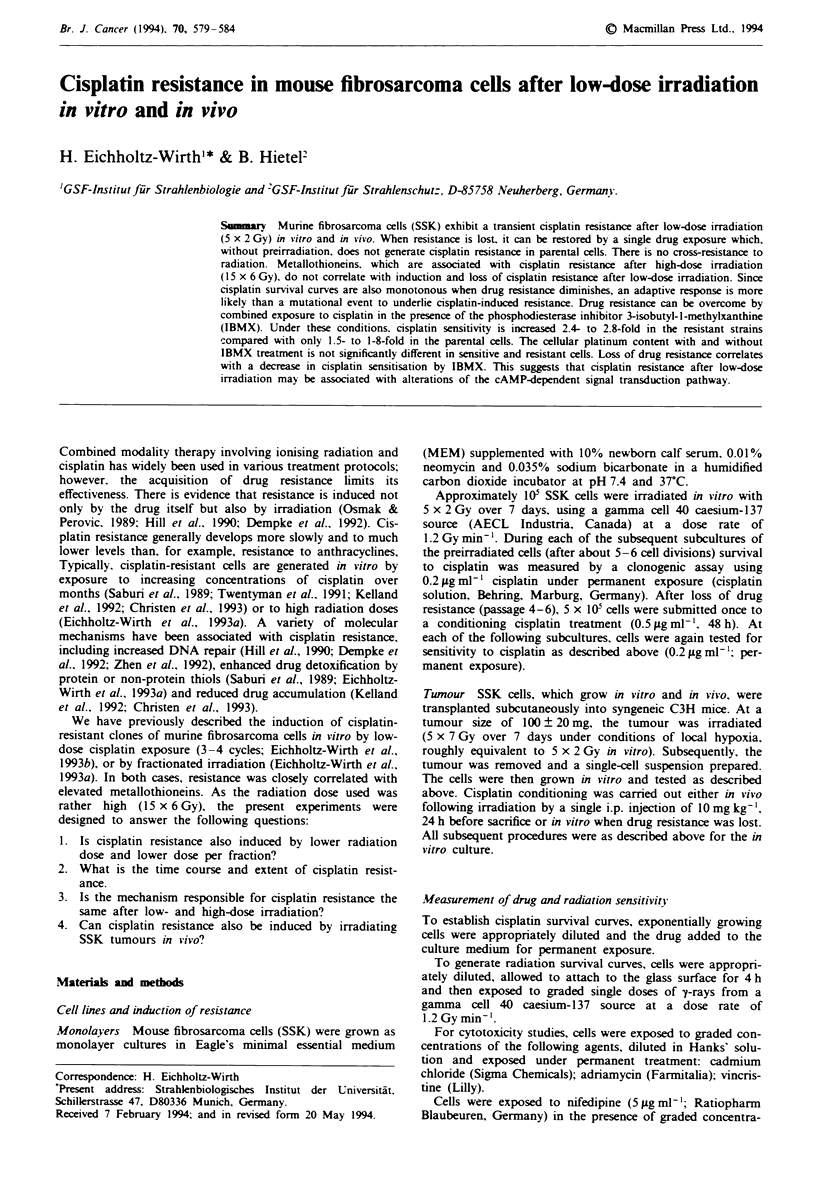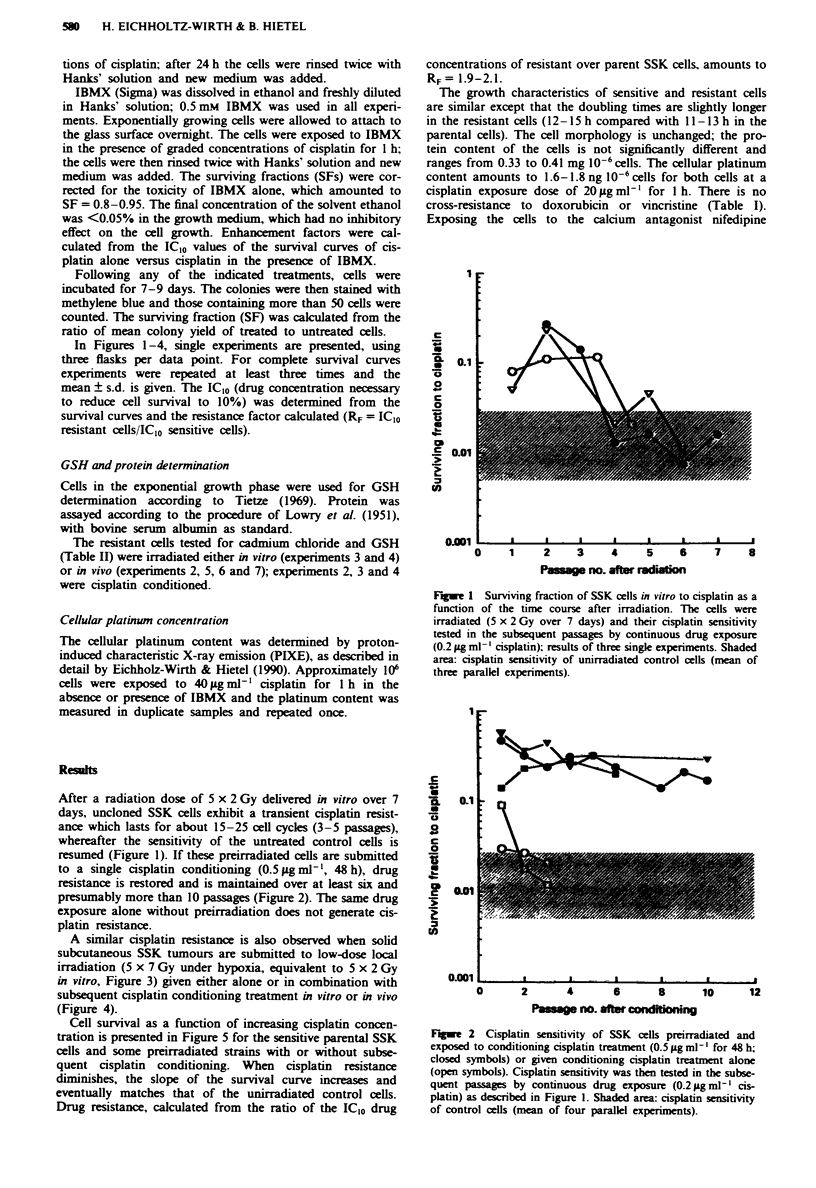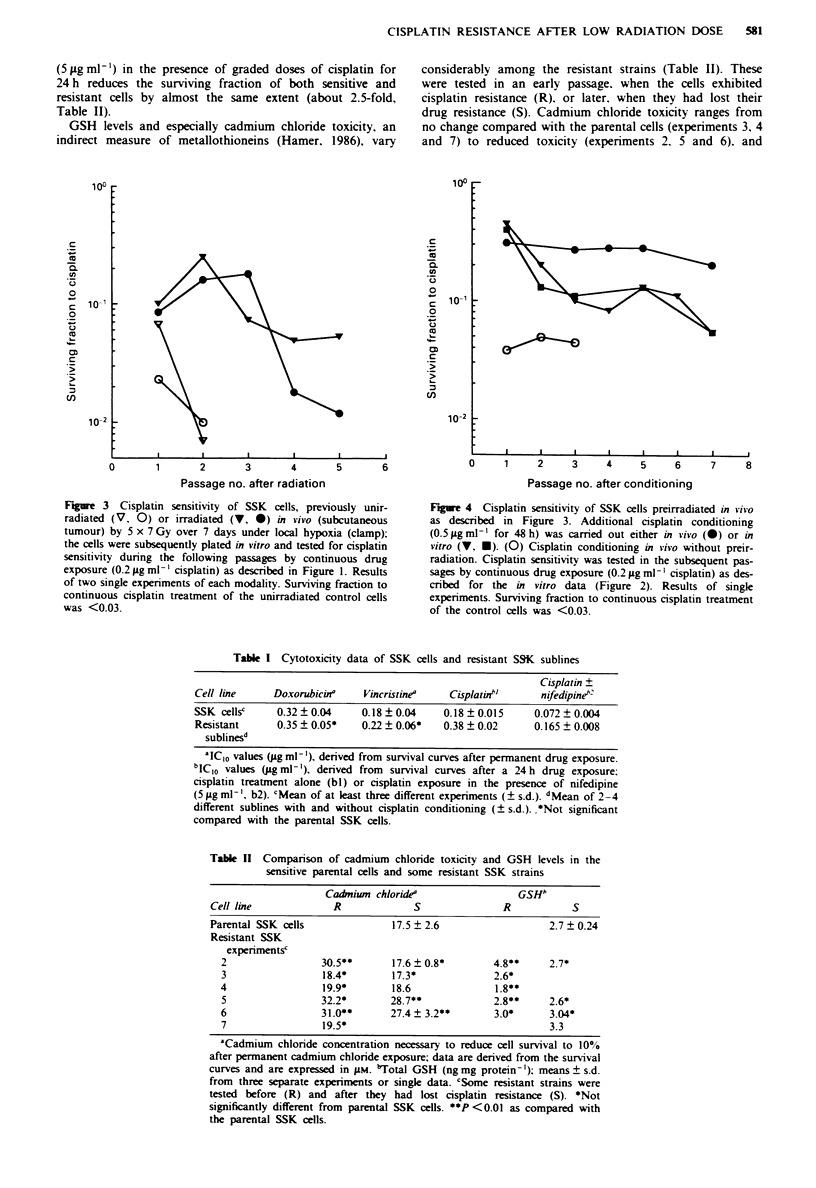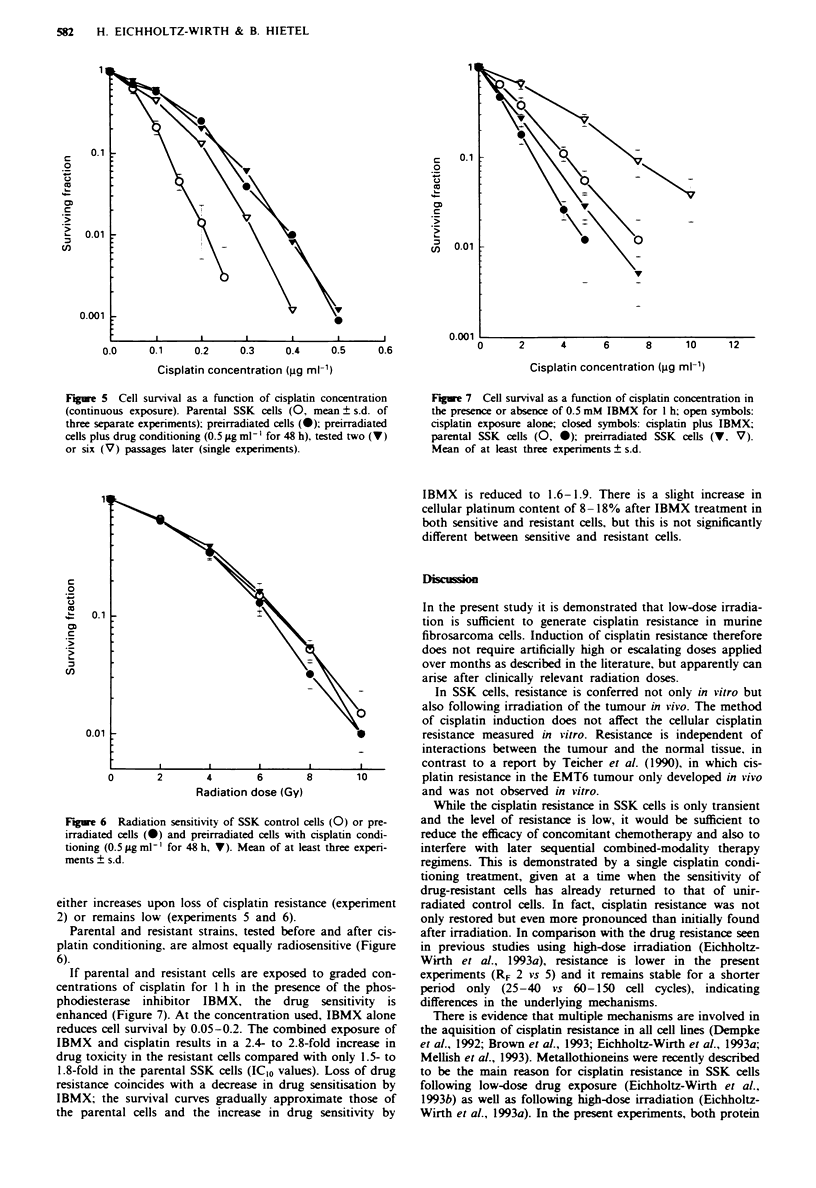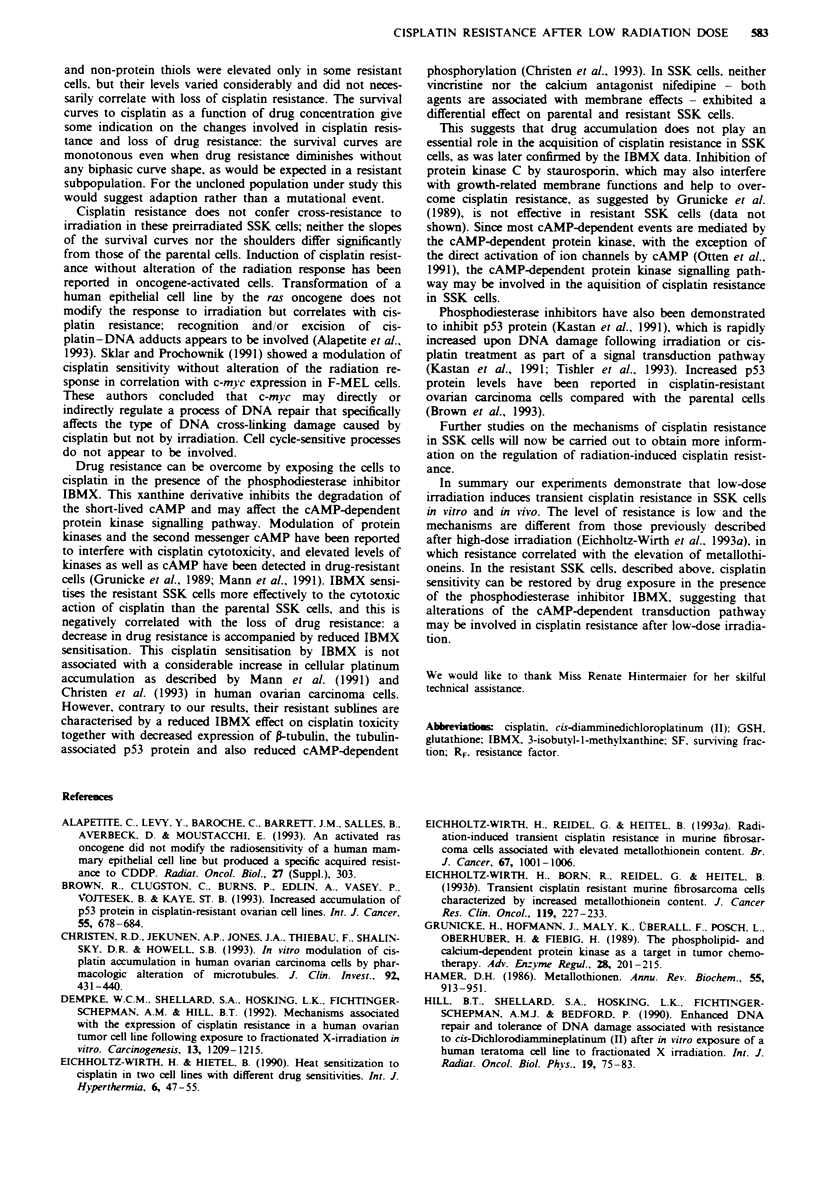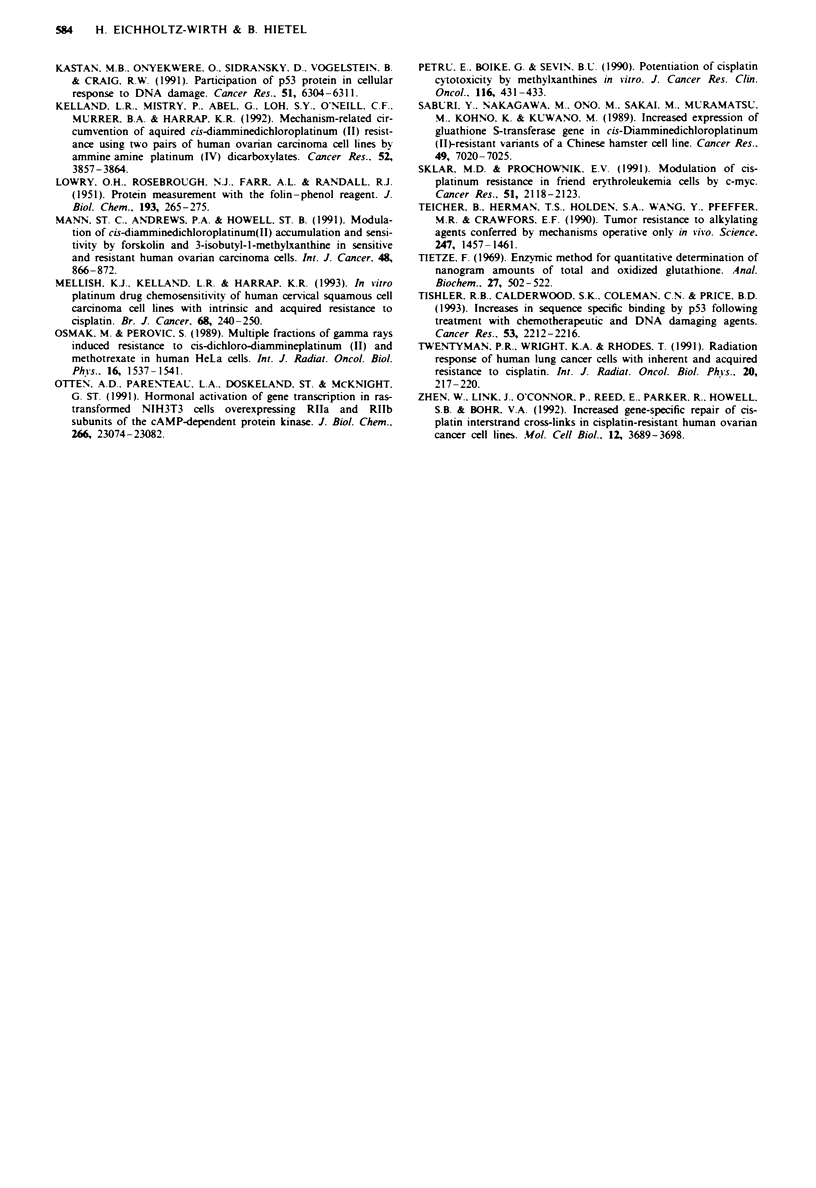# Cisplatin resistance in mouse fibrosarcoma cells after low-dose irradiation in vitro and in vivo.

**DOI:** 10.1038/bjc.1994.354

**Published:** 1994-10

**Authors:** H. Eichholtz-Wirth, B. Hietel

**Affiliations:** GSF-Institut für Strahlenbiologie, Neuherberg, Germany.

## Abstract

Murine fibrosarcoma cells (SSK) exhibit a transient cisplatin resistance after low-dose irradiation (5 x 2 Gy) in vitro and in vivo. When resistance is lost, it can be restored by a single drug exposure which, without preirradiation, does not generate cisplatin resistance in parental cells. There is no cross-resistance to radiation. Metallothioneins, which are associated with cisplatin resistance after high-dose irradiation (15 x 6 Gy), do not correlate with induction and loss of cisplatin resistance after low-dose irradiation. Since cisplatin survival curves are also monotonous when drug resistance diminishes, an adaptive response is more likely than a mutational event to underlie cisplatin-induced resistance. Drug resistance can be overcome by combined exposure to cisplatin in the presence of the phosphodiesterase inhibitor 3-isobutyl-1-methylxanthine (IBMX). Under these conditions, cisplatin sensitivity is increased 2.4- to 2.8-fold in the resistant strains compared with only 1.5- to 1.8-fold in the parental cells. The cellular platinum content with and without IBMX treatment is not significantly different in sensitive and resistant cells. Loss of drug resistance correlates with a decrease in cisplatin sensitisation by IBMX. This suggests that cisplatin resistance after low-dose irradiation may be associated with alterations of the cAMP-dependent signal transduction pathway.


*Br. J. Cancer* (1994), **70**, 579–584

© Macmillan Press Ltd., 1994

### Cisplatin resistance in mouse fibrosarcoma cells after low-dose irradiation *in vitro* and *in vivo*

H. Eichholtz-Wirth[1]* & B. Hietel[2]

[1]*GSF-Institut für Strahlenbiologie and* [2]*GSF-Institut für Strahlenschutz, D-85758 Neuherberg, Germany.*

**Summary** Murine fibrosarcoma cells (SSK) exhibit a transient cisplatin resistance after low-dose irradiation (5 × 2 Gy) *in vitro* and *in vivo*. When resistance is lost, it can be restored by a single drug exposure which, without preirradiation, does not generate cisplatin resistance in parental cells. There is no cross-resistance to radiation. Metallothioneins, which are associated with cisplatin resistance after high-dose irradiation (15 × 6 Gy), do not correlate with induction and loss of cisplatin resistance after low-dose irradiation. Since cisplatin survival curves are also monotonous when drug resistance diminishes, an adaptive response is more likely than a mutational event to underlie cisplatin-induced resistance. Drug resistance can be overcome by combined exposure to cisplatin in the presence of the phosphodiesterase inhibitor 3-isobutyl-1-methylxanthine (IBMX). Under these conditions, cisplatin sensitivity is increased 2.4- to 2.8-fold in the resistant strains compared with only 1.5- to 1-8-fold in the parental cells. The cellular platinum content with and without IBMX treatment is not significantly different in sensitive and resistant cells. Loss of drug resistance correlates with a decrease in cisplatin sensitisation by IBMX. This suggests that cisplatin resistance after low-dose irradiation may be associated with alterations of the cAMP-dependent signal transduction pathway.

Combined modality therapy involving ionising radiation and cisplatin has widely been used in various treatment protocols; however, the acquisition of drug resistance limits its effectiveness. There is evidence that resistance is induced not only by the drug itself but also by irradiation (Osmak & Perovic, 1989; Hill *et al*., 1990; Dempke *et al*., 1992). Cisplatin resistance generally develops more slowly and to much lower levels than, for example, resistance to anthracyclines, Typically, cisplatin-resistant cells are generated *in vitro* by exposure to increasing concentrations of cisplatin over months (Saburi *et al*., 1989; Twentyman *et al*., 1991; Kelland *et al*., 1992; Christen *et al*., 1993) or to high radiation doses (Eichholtz-Wirth *et al*., 1993*a*). A variety of molecular mechanisms have been associated with cisplatin resistance, including increased DNA repair (Hill *et al*., 1990; Dempke *et al*., 1992; Zhen *et al*., 1992), enhanced drug detoxification by protein or non-protein thiols (Saburi *et al*., 1989; Eichholtz-Wirth *et al*., 1993*a*) and reduced drug accumulation (Kelland *et al*., 1992; Christen *et al*., 1993).

We have previously described the induction of cisplatin-resistant clones of murine fibrosarcoma cells *in vitro* by low-dose cisplatin exposure (3–4 cycles; Eichholtz-Wirth *et al*., 1993*b*), or by fractionated irradiation (Eichholtz-Wirth *et al*., 1993*a*). In both cases, resistance was closely correlated with elevated metallothioneins. As the radiation dose used was rather high (15 × 6 Gy), the present experiments were designed to answer the following questions:

1. Is cisplatin resistance also induced by lower radiation dose and lower dose per fraction?
2. What is the time course and extent of cisplatin resistance.
3. Is the mechanism responsible for cisplatin resistance the same after low- and high-dose irradiation?
4. Can cisplatin resistance also be induced by irradiating SSK tumours *in vivo*?

### Materials and methods

### *Cell lines and induction of resistance*

*Monolayers* Mouse fibrosarcoma cells (SSK) were grown as monolayer cultures in Eagle's minimal essential medium (MEM) supplemented with 10% newborn calf serum, 0.01% neomycin and 0.035% sodium bicarbonate in a humidified carbon dioxide incubator at pH 7.4 and 37°C.

Approximately $10^5$ SSK cells were irradiated *in vitro* with 5 × 2 Gy over 7 days, using a gamma cell 40 caesium-137 source (AECL Industria, Canada) at a dose rate of 1.2 Gy $min^{-1}$. During each of the subsequent subcultures of the preirradiated cells (after about 5–6 cell divisions) survival to cisplatin was measured by a clonogenic assay using 0.2 $\mu g\ ml^{-1}$ cisplatin under permanent exposure (cisplatin solution, Behring, Marburg, Germany). After loss of drug resistance (passage 4–6), 5 × $10^5$ cells were submitted once to a conditioning cisplatin treatment (0.5 $\mu g\ ml^{-1}$, 48 h). At each of the following subcultures, cells were again tested for sensitivity to cisplatin as described above (0.2 $\mu g\ ml^{-1}$; permanent exposure).

*Tumour* SSK cells, which grow *in vitro* and *in vivo*, were transplanted subcutaneously into syngeneic C3H mice. At a tumour size of 100 ± 20 mg, the tumour was irradiated (5 × 7 Gy over 7 days under conditions of local hypoxia, roughly equivalent to 5 × 2 Gy *in vitro*). Subsequently, the tumour was removed and a single-cell suspension prepared. The cells were then grown *in vitro* and tested as described above. Cisplatin conditioning was carried out either *in vivo* following irradiation by a single i.p. injection of 10 mg $kg^{-1}$, 24 h before sacrifice or *in vitro* when drug resistance was lost. All subsequent procedures were as described above for the *in vitro* culture.

### *Measurement of drug and radiation sensitivity*

To establish cisplatin survival curves, exponentially growing cells were appropriately diluted and the drug added to the culture medium for permanent exposure.

To generate radiation survival curves, cells were appropriately diluted, allowed to attach to the glass surface for 4 h and then exposed to graded single doses of γ-rays from a gamma cell 40 caesium-137 source at a dose rate of 1.2 Gy $min^{-1}$.

For cytotoxicity studies, cells were exposed to graded concentrations of the following agents, diluted in Hanks' solution and exposed under permanent treatment: cadmium chloride (Sigma Chemicals); adriamycin (Farmitalia); vincristine (Lilly).

Cells were exposed to nifedipine (5 $\mu g\ ml^{-1}$; Ratiopharm Blaubeuren, Germany) in the presence of graded concentra-

Correspondence: H. Eichholtz-Wirth
*Present address: Strahlenbiologisches Institut der Universität, Schillerstrasse 47, D80336 Munich, Germany.
Received 7 February 1994; and in revised form 20 May 1994.

tions of cisplatin; after 24 h the cells were rinsed twice with Hanks' solution and new medium was added.

IBMX (Sigma) was dissolved in ethanol and freshly diluted in Hanks' solution; 0.5 mM IBMX was used in all experiments. Exponentially growing cells were allowed to attach to the glass surface overnight. The cells were exposed to IBMX in the presence of graded concentrations of cisplatin for 1 h; the cells were then rinsed twice with Hanks' solution and new medium was added. The surviving fractions (SFs) were corrected for the toxicity of IBMX alone, which amounted to SF = 0.8–0.95. The final concentration of the solvent ethanol was <0.05% in the growth medium, which had no inhibitory effect on the cell growth. Enhancement factors were calculated from the $IC_{10}$ values of the survival curves of cisplatin alone versus cisplatin in the presence of IBMX.

Following any of the indicated treatments, cells were incubated for 7–9 days. The colonies were then stained with methylene blue and those containing more than 50 cells were counted. The surviving fraction (SF) was calculated from the ratio of mean colony yield of treated to untreated cells.

In Figures 1–4, single experiments are presented, using three flasks per data point. For complete survival curves experiments were repeated at least three times and the mean $\pm$ s.d. is given. The $IC_{10}$ (drug concentration necessary to reduce cell survival to 10%) was determined from the survival curves and the resistance factor calculated ($R_F = IC_{10}$ resistant cells/$IC_{10}$ sensitive cells).

### *GSH and protein determination*

Cells in the exponential growth phase were used for GSH determination according to Tietze (1969). Protein was assayed according to the procedure of Lowry *et al.* (1951), with bovine serum albumin as standard.

The resistant cells tested for cadmium chloride and GSH (Table II) were irradiated either *in vitro* (experiments 3 and 4) or *in vivo* (experiments 2, 5, 6 and 7); experiments 2, 3 and 4 were cisplatin conditioned.

### *Cellular platinum concentration*

The cellular platinum content was determined by proton-induced characteristic X-ray emission (PIXE), as described in detail by Eichholz-Wirth & Hietel (1990). Approximately $10^6$ cells were exposed to 40 $\mu g\ ml^{-1}$ cisplatin for 1 h in the absence or presence of IBMX and the platinum content was measured in duplicate samples and repeated once.

### Results

After a radiation dose of 5 × 2 Gy delivered *in vitro* over 7 days, uncloned SSK cells exhibit a transient cisplatin resistance which lasts for about 15–25 cell cycles (3–5 passages), whereafter the sensitivity of the untreated control cells is resumed (Figure 1). If these preirradiated cells are submitted to a single cisplatin conditioning (0.5 $\mu g\ ml^{-1}$, 48 h), drug resistance is restored and is maintained over at least six and presumably more than 10 passages (Figure 2). The same drug exposure alone without preirradiation does not generate cisplatin resistance.

A similar cisplatin resistance is also observed when solid subcutaneous SSK tumours are submitted to low-dose local irradiation (5 × 7 Gy under hypoxia, equivalent to 5 × 2 Gy *in vitro*, Figure 3) given either alone or in combination with subsequent cisplatin conditioning treatment *in vitro* or *in vivo* (Figure 4).

Cell survival as a function of increasing cisplatin concentration is presented in Figure 5 for the sensitive parental SSK cells and some preirradiated strains with or without subsequent cisplatin conditioning. When cisplatin resistance diminishes, the slope of the survival curve increases and eventually matches that of the unirradiated control cells. Drug resistance, calculated from the ratio of the $IC_{10}$ drug concentrations of resistant over parent SSK cells, amounts to $R_F = 1.9–2.1$.

The growth characteristics of sensitive and resistant cells are similar except that the doubling times are slightly longer in the resistant cells (12–15 h compared with 11–13 h in the parental cells). The cell morphology is unchanged; the protein content of the cells is not significantly different and ranges from 0.33 to 0.41 mg $10^{-6}$ cells. The cellular platinum content amounts to 1.6–1.8 ng $10^{-6}$ cells for both cells at a cisplatin exposure dose of 20 $\mu g\ ml^{-1}$ for 1 h. There is no cross-resistance to doxorubicin or vincristine (Table I). Exposing the cells to the calcium antagonist nifedipine

**Figure 1** Surviving fraction of SSK cells *in vitro* to cisplatin as a function of the time course after irradiation. The cells were irradiated (5 × 2 Gy over 7 days) and their cisplatin sensitivity tested in the subsequent passages by continuous drug exposure (0.2 $\mu g\ ml^{-1}$ cisplatin); results of three single experiments. Shaded area: cisplatin sensitivity of unirradiated control cells (mean of three parallel experiments).

**Figure 2** Cisplatin sensitivity of SSK cells preirradiated and exposed to conditioning cisplatin treatment (0.5 $\mu g\ ml^{-1}$ for 48 h; closed symbols) or given conditioning cisplatin treatment alone (open symbols). Cisplatin sensitivity was then tested in the subsequent passages by continuous drug exposure (0.2 $\mu g\ ml^{-1}$ cisplatin) as described in Figure 1. Shaded area: cisplatin sensitivity of control cells (mean of four parallel experiments).

($5\,\mu g\,ml^{-1}$) in the presence of graded doses of cisplatin for 24 h reduces the surviving fraction of both sensitive and resistant cells by almost the same extent (about 2.5-fold, Table II).

GSH levels and especially cadmium chloride toxicity, an indirect measure of metallothioneins (Hamer, 1986), vary considerably among the resistant strains (Table II). These were tested in an early passage, when the cells exhibited cisplatin resistance (R), or later, when they had lost their drug resistance (S). Cadmium chloride toxicity ranges from no change compared with the parental cells (experiments 3, 4 and 7) to reduced toxicity (experiments 2, 5 and 6), and

**Figure 3** Cisplatin sensitivity of SSK cells, previously unirradiated (▽, ○) or irradiated (▼, ●) *in vivo* (subcutaneous tumour) by 5 × 7 Gy over 7 days under local hypoxia (clamp); the cells were subsequently plated *in vitro* and tested for cisplatin sensitivity during the following passages by continuous drug exposure ($0.2\,\mu g\,ml^{-1}$ cisplatin) as described in Figure 1. Results of two single experiments of each modality. Surviving fraction to continuous cisplatin treatment of the unirradiated control cells was <0.03.

**Figure 4** Cisplatin sensitivity of SSK cells preirradiated *in vivo* as described in Figure 3. Additional cisplatin conditioning ($0.5\,\mu g\,ml^{-1}$ for 48 h) was carried out either *in vivo* (●) or *in vitro* (▼, ■). (○) Cisplatin conditioning *in vivo* without preirradiation. Cisplatin sensitivity was tested in the subsequent passages by continuous drug exposure ($0.2\,\mu g\,ml^{-1}$ cisplatin) as described for the *in vitro* data (Figure 2). Results of single experiments. Surviving fraction to continuous cisplatin treatment of the control cells was <0.03.

**Table I** Cytotoxicity data of SSK cells and resistant SSK sublines

| *Cell line* | *Doxorubicin*[a] | *Vincristine*[a] | *Cisplatin*[b1] | *Cisplatin* ± *nifedipine*[b2] |
|---|---|---|---|---|
| SSK cells[c] | 0.32 ± 0.04 | 0.18 ± 0.04 | 0.18 ± 0.015 | 0.072 ± 0.004 |
| Resistant sublines[d] | 0.35 ± 0.05* | 0.22 ± 0.06* | 0.38 ± 0.02 | 0.165 ± 0.008 |

[a]$IC_{10}$ values ($\mu g\,ml^{-1}$), derived from survival curves after permanent drug exposure. [b]$IC_{10}$ values ($\mu g\,ml^{-1}$), derived from survival curves after a 24 h drug exposure; cisplatin treatment alone (b1) or cisplatin exposure in the presence of nifedipine ($5\,\mu g\,ml^{-1}$, b2). [c]Mean of at least three different experiments (± s.d.). [d]Mean of 2–4 different sublines with and without cisplatin conditioning (± s.d.). .*Not significant compared with the parental SSK cells.

**Table II** Comparison of cadmium chloride toxicity and GSH levels in the sensitive parental cells and some resistant SSK strains

| *Cell line* | *Cadmium chloride*[a] *R* | *S* | *GSH*[b] *R* | *S* |
|---|---|---|---|---|
| Parental SSK cells | | 17.5 ± 2.6 | | 2.7 ± 0.24 |
| Resistant SSK experiments[c] | | | | |
| 2 | 30.5** | 17.6 ± 0.8* | 4.8** | 2.7* |
| 3 | 18.4* | 17.3* | 2.6* | |
| 4 | 19.9* | 18.6 | 1.8** | |
| 5 | 32.2* | 28.7** | 2.8** | 2.6* |
| 6 | 31.0** | 27.4 ± 3.2** | 3.0* | 3.04* |
| 7 | 19.5* | | | 3.3 |

[a]Cadmium chloride concentration necessary to reduce cell survival to 10% after permanent cadmium chloride exposure; data are derived from the survival curves and are expressed in µM. [b]Total GSH (ng mg protein$^{-1}$); means ± s.d. from three separate experiments or single data. [c]Some resistant strains were tested before (R) and after they had lost cisplatin resistance (S). *Not significantly different from parental SSK cells. **$P < 0.01$ as compared with the parental SSK cells.

**Figure 5** Cell survival as a function of cisplatin concentration (continuous exposure). Parental SSK cells (○, mean ± s.d. of three separate experiments); preirradiated cells (●); preirradiated cells plus drug conditioning (0.5 μg ml$^{-1}$ for 48 h), tested two (▼) or six (▽) passages later (single experiments).

**Figure 7** Cell survival as a function of cisplatin concentration in the presence or absence of 0.5 mM IBMX for 1 h; open symbols: cisplatin exposure alone; closed symbols: cisplatin plus IBMX; parental SSK cells (○, ●); preirradiated SSK cells (▼, ▽). Mean of at least three experiments ± s.d.

**Figure 6** Radiation sensitivity of SSK control cells (○) or preirradiated cells (●) and preirradiated cells with cisplatin conditioning (0.5 μg ml$^{-1}$ for 48 h, ▼). Mean of at least three experiments ± s.d.

either increases upon loss of cisplatin resistance (experiment 2) or remains low (experiments 5 and 6).

Parental and resistant strains, tested before and after cisplatin conditioning, are almost equally radiosensitive (Figure 6).

If parental and resistant cells are exposed to graded concentrations of cisplatin for 1 h in the presence of the phosphodiesterase inhibitor IBMX, the drug sensitivity is enhanced (Figure 7). At the concentration used, IBMX alone reduces cell survival by 0.05–0.2. The combined exposure of IBMX and cisplatin results in a 2.4- to 2.8-fold increase in drug toxicity in the resistant cells compared with only 1.5- to 1.8-fold in the parental SSK cells ($IC_{10}$ values). Loss of drug resistance coincides with a decrease in drug sensitisation by IBMX; the survival curves gradually approximate those of the parental cells and the increase in drug sensitivity by IBMX is reduced to 1.6–1.9. There is a slight increase in cellular platinum content of 8–18% after IBMX treatment in both sensitive and resistant cells, but this is not significantly different between sensitive and resistant cells.

### Discussion

In the present study it is demonstrated that low-dose irradiation is sufficient to generate cisplatin resistance in murine fibrosarcoma cells. Induction of cisplatin resistance therefore does not require artificially high or escalating doses applied over months as described in the literature, but apparently can arise after clinically relevant radiation doses.

In SSK cells, resistance is conferred not only *in vitro* but also following irradiation of the tumour *in vivo*. The method of cisplatin induction does not affect the cellular cisplatin resistance measured *in vitro*. Resistance is independent of interactions between the tumour and the normal tissue, in contrast to a report by Teicher *et al.* (1990), in which cisplatin resistance in the EMT6 tumour only developed *in vivo* and was not observed *in vitro*.

While the cisplatin resistance in SSK cells is only transient and the level of resistance is low, it would be sufficient to reduce the efficacy of concomitant chemotherapy and also to interfere with later sequential combined-modality therapy regimens. This is demonstrated by a single cisplatin conditioning treatment, given at a time when the sensitivity of drug-resistant cells has already returned to that of unirradiated control cells. In fact, cisplatin resistance was not only restored but even more pronounced than initially found after irradiation. In comparison with the drug resistance seen in previous studies using high-dose irradiation (Eichholtz-Wirth *et al.*, 1993*a*), resistance is lower in the present experiments ($R_F$ 2 *vs* 5) and it remains stable for a shorter period only (25–40 *vs* 60–150 cell cycles), indicating differences in the underlying mechanisms.

There is evidence that multiple mechanisms are involved in the aquisition of cisplatin resistance in all cell lines (Dempke *et al.*, 1992; Brown *et al.*, 1993; Eichholtz-Wirth *et al.*, 1993*a*; Mellish *et al.*, 1993). Metallothioneins were recently described to be the main reason for cisplatin resistance in SSK cells following low-dose drug exposure (Eichholtz-Wirth *et al.*, 1993*b*) as well as following high-dose irradiation (Eichholtz-Wirth *et al.*, 1993*a*). In the present experiments, both protein and non-protein thiols were elevated only in some resistant cells, but their levels varied considerably and did not necessarily correlate with loss of cisplatin resistance. The survival curves to cisplatin as a function of drug concentration give some indication on the changes involved in cisplatin resistance and loss of drug resistance: the survival curves are monotonous even when drug resistance diminishes without any biphasic curve shape, as would be expected in a resistant subpopulation. For the uncloned population under study this would suggest adaption rather than a mutational event.

Cisplatin resistance does not confer cross-resistance to irradiation in these preirradiated SSK cells; neither the slopes of the survival curves nor the shoulders differ significantly from those of the parental cells. Induction of cisplatin resistance without alteration of the radiation response has been reported in oncogene-activated cells. Transformation of a human epithelial cell line by the *ras* oncogene does not modify the response to irradiation but correlates with cisplatin resistance; recognition and/or excision of cisplatin–DNA adducts appears to be involved (Alapetite *et al.*, 1993). Sklar and Prochownik (1991) showed a modulation of cisplatin sensitivity without alteration of the radiation response in correlation with c-*myc* expression in F-MEL cells. These authors concluded that c-*myc* may directly or indirectly regulate a process of DNA repair that specifically affects the type of DNA cross-linking damage caused by cisplatin but not by irradiation. Cell cycle-sensitive processes do not appear to be involved.

Drug resistance can be overcome by exposing the cells to cisplatin in the presence of the phosphodiesterase inhibitor IBMX. This xanthine derivative inhibits the degradation of the short-lived cAMP and may affect the cAMP-dependent protein kinase signalling pathway. Modulation of protein kinases and the second messenger cAMP have been reported to interfere with cisplatin cytotoxicity, and elevated levels of kinases as well as cAMP have been detected in drug-resistant cells (Grunicke *et al.*, 1989; Mann *et al.*, 1991). IBMX sensitises the resistant SSK cells more effectively to the cytotoxic action of cisplatin than the parental SSK cells, and this is negatively correlated with the loss of drug resistance: a decrease in drug resistance is accompanied by reduced IBMX sensitisation. This cisplatin sensitisation by IBMX is not associated with a considerable increase in cellular platinum accumulation as described by Mann *et al.* (1991) and Christen *et al.* (1993) in human ovarian carcinoma cells. However, contrary to our results, their resistant sublines are characterised by a reduced IBMX effect on cisplatin toxicity together with decreased expression of $\beta$-tubulin, the tubulin-associated p53 protein and also reduced cAMP-dependent phosphorylation (Christen *et al.*, 1993). In SSK cells, neither vincristine nor the calcium antagonist nifedipine – both agents are associated with membrane effects – exhibited a differential effect on parental and resistant SSK cells.

This suggests that drug accumulation does not play an essential role in the acquisition of cisplatin resistance in SSK cells, as was later confirmed by the IBMX data. Inhibition of protein kinase C by staurosporin, which may also interfere with growth-related membrane functions and help to overcome cisplatin resistance, as suggested by Grunicke *et al.* (1989), is not effective in resistant SSK cells (data not shown). Since most cAMP-dependent events are mediated by the cAMP-dependent protein kinase, with the exception of the direct activation of ion channels by cAMP (Otten *et al.*, 1991), the cAMP-dependent protein kinase signalling pathway may be involved in the aquisition of cisplatin resistance in SSK cells.

Phosphodiesterase inhibitors have also been demonstrated to inhibit p53 protein (Kastan *et al.*, 1991), which is rapidly increased upon DNA damage following irradiation or cisplatin treatment as part of a signal transduction pathway (Kastan *et al.*, 1991; Tishler *et al.*, 1993). Increased p53 protein levels have been reported in cisplatin-resistant ovarian carcinoma cells compared with the parental cells (Brown *et al.*, 1993).

Further studies on the mechanisms of cisplatin resistance in SSK cells will now be carried out to obtain more information on the regulation of radiation-induced cisplatin resistance.

In summary our experiments demonstrate that low-dose irradiation induces transient cisplatin resistance in SSK cells *in vitro* and *in vivo*. The level of resistance is low and the mechanisms are different from those previously described after high-dose irradiation (Eichholtz-Wirth *et al.*, 1993*a*), in which resistance correlated with the elevation of metallothioneins. In the resistant SSK cells, described above, cisplatin sensitivity can be restored by drug exposure in the presence of the phosphodiesterase inhibitor IBMX, suggesting that alterations of the cAMP-dependent transduction pathway may be involved in cisplatin resistance after low-dose irradiation.

We would like to thank Miss Renate Hintermaier for her skilful technical assistance.

**Abbreviations:** cisplatin, *cis*-diamminedichloroplatinum (II); GSH, glutathione; IBMX, 3-isobutyl-1-methylxanthine; SF, surviving fraction; $R_F$, resistance factor.

**References**

ALAPETITE, C., LEVY, Y., BAROCHE, C., BARRETT, J.M., SALLES, B., AVERBECK, D. & MOUSTACCHI, E. (1993). An activated ras oncogene did not modify the radiosensitivity of a human mammary epithelial cell line but produced a specific acquired resistance to CDDP. *Radiat. Oncol. Biol.*, **27** (Suppl.), 303.

BROWN, R., CLUGSTON, C., BURNS, P., EDLIN, A., VASEY, P., VOJTESEK, B. & KAYE, ST. B. (1993). Increased accumulation of p53 protein in cisplatin-resistant ovarian cell lines. *Int. J. Cancer*, **55**, 678–684.

CHRISTEN, R.D., JEKUNEN, A.P., JONES, J.A., THIEBAU, F., SHALINSKY, D.R. & HOWELL, S.B. (1993). *In vitro* modulation of cisplatin accumulation in human ovarian carcinoma cells by pharmacologic alteration of microtubules. *J. Clin. Invest.*, **92**, 431–440.

DEMPKE, W.C.M., SHELLARD, S.A., HOSKING, L.K., FICHTINGER-SCHEPMAN, A.M. & HILL, B.T. (1992). Mechanisms associated with the expression of cisplatin resistance in a human ovarian tumor cell line following exposure to fractionated X-irradiation *in vitro*. *Carcinogenesis*, **13**, 1209–1215.

EICHHOLTZ-WIRTH, H. & HIETEL, B. (1990). Heat sensitization to cisplatin in two cell lines with different drug sensitivities. *Int. J. Hyperthermia*, **6**, 47–55.

EICHHOLTZ-WIRTH, H., REIDEL, G. & HEITEL, B. (1993*a*). Radiation-induced transient cisplatin resistance in murine fibrosarcoma cells associated with elevated metallothionein content. *Br. J. Cancer*, **67**, 1001–1006.

EICHHOLTZ-WIRTH, H., BORN, R., REIDEL, G. & HEITEL, B. (1993*b*). Transient cisplatin resistant murine fibrosarcoma cells characterized by increased metallothionein content. *J. Cancer Res. Clin. Oncol.*, **119**, 227–233.

GRUNICKE, H., HOFMANN, J., MALY, K., ÜBERALL, F., POSCH, L., OBERHUBER, H. & FIEBIG, H. (1989). The phospholipid- and calcium-dependent protein kinase as a target in tumor chemotherapy. *Adv. Enzyme Regul.*, **28**, 201–215.

HAMER, D.H. (1986). Metallothionen. *Annu. Rev. Biochem.*, **55**, 913–951.

HILL, B.T., SHELLARD, S.A., HOSKING, L.K., FICHTINGER-SCHEPMAN, A.M.J. & BEDFORD, P. (1990). Enhanced DNA repair and tolerance of DNA damage associated with resistance to *cis*-Dichlorodiammineplatinum (II) after *in vitro* exposure of a human teratoma cell line to fractionated X irradiation. *Int. J. Radiat. Oncol. Biol. Phys.*, **19**, 75–83.

KASTAN, M.B., ONYEKWERE, O., SIDRANSKY, D., VOGELSTEIN, B. & CRAIG, R.W. (1991). Participation of p53 protein in cellular response to DNA damage. *Cancer Res.*, **51**, 6304-6311.

KELLAND, L.R., MISTRY, P., ABEL, G., LOH, S.Y., O'NEILL, C.F., MURRER, B.A. & HARRAP, K.R. (1992). Mechanism-related circumvention of aquired *cis*-diamminedichloroplatinum (II) resistance using two pairs of human ovarian carcinoma cell lines by ammine amine platinum (IV) dicarboxylates. *Cancer Res.*, **52**, 3857-3864.

LOWRY, O.H., ROSEBROUGH, N.J., FARR, A.L. & RANDALL, R.J. (1951). Protein measurement with the folin-phenol reagent. *J. Biol. Chem.*, **193**, 265-275.

MANN, ST. C., ANDREWS, P.A. & HOWELL, ST. B. (1991). Modulation of *cis*-diamminedichloroplatinum(II) accumulation and sensitivity by forskolin and 3-isobutyl-1-methylxanthine in sensitive and resistant human ovarian carcinoma cells. *Int. J. Cancer*, **48**, 866-872.

MELLISH, K.J., KELLAND, L.R. & HARRAP, K.R. (1993). *In vitro* platinum drug chemosensitivity of human cervical squamous cell carcinoma cell lines with intrinsic and acquired resistance to cisplatin. *Br. J. Cancer*, **68**, 240-250.

OSMAK, M. & PEROVIC, S. (1989). Multiple fractions of gamma rays induced resistance to cis-dichloro-diammineplatinum (II) and methotrexate in human HeLa cells. *Int. J. Radiat. Oncol. Biol. Phys.*, **16**, 1537-1541.

OTTEN, A.D., PARENTEAU, L.A., DOSKELAND, ST. & MCKNIGHT, G. ST. (1991). Hormonal activation of gene transcription in ras-transformed NIH3T3 cells overexpressing RIIa and RIIb subunits of the cAMP-dependent protein kinase. *J. Biol. Chem.*, **266**, 23074-23082.

PETRU, E., BOIKE, G. & SEVIN, B.U. (1990). Potentiation of cisplatin cytotoxicity by methylxanthines *in vitro*. *J. Cancer Res. Clin. Oncol.*, **116**, 431-433.

SABURI, Y., NAKAGAWA, M., ONO, M., SAKAI, M., MURAMATSU, M., KOHNO, K. & KUWANO, M. (1989). Increased expression of gluathione S-transferase gene in *cis*-Diamminedichloroplatinum (II)-resistant variants of a Chinese hamster cell line. *Cancer Res.*, **49**, 7020-7025.

SKLAR, M.D. & PROCHOWNIK, E.V. (1991). Modulation of cis-platinum resistance in friend erythroleukemia cells by c-myc. *Cancer Res.*, **51**, 2118-2123.

TEICHER, B., HERMAN, T.S., HOLDEN, S.A., WANG, Y., PFEFFER, M.R. & CRAWFORS, E.F. (1990). Tumor resistance to alkylating agents conferred by mechanisms operative only *in vivo*. *Science*, **247**, 1457-1461.

TIETZE, F. (1969). Enzymic method for quantitative determination of nanogram amounts of total and oxidized glutathione. *Anal. Biochem.*, **27**, 502-522.

TISHLER, R.B., CALDERWOOD, S.K., COLEMAN, C.N. & PRICE, B.D. (1993). Increases in sequence specific binding by p53 following treatment with chemotherapeutic and DNA damaging agents. *Cancer Res.*, **53**, 2212-2216.

TWENTYMAN, P.R., WRIGHT, K.A. & RHODES, T. (1991). Radiation response of human lung cancer cells with inherent and acquired resistance to cisplatin. *Int. J. Radiat. Oncol. Biol. Phys.*, **20**, 217-220.

ZHEN, W., LINK, J., O'CONNOR, P., REED, E., PARKER, R., HOWELL, S.B. & BOHR, V.A. (1992). Increased gene-specific repair of cisplatin interstrand cross-links in cisplatin-resistant human ovarian cancer cell lines. *Mol. Cell Biol.*, **12**, 3689-3698.